# Supplementary material for: Impact of diet and host genetics on the murine intestinal mycobiome
Source: Nat Commun. 2023 Feb 14;14:834. doi: 10.1038/s41467-023-36479-z (PMC9929102; doi:10.1038/s41467-023-36479-z)
Supplement: Supplementary file 1 — Supplementary Information [file 41467_2023_36479_MOESM1_ESM.pdf]

Supplementary Information

for

Impact of diet and host genetics on the murine intestinal mycobiome

Gupta, Ernst & Vorobyev et al.

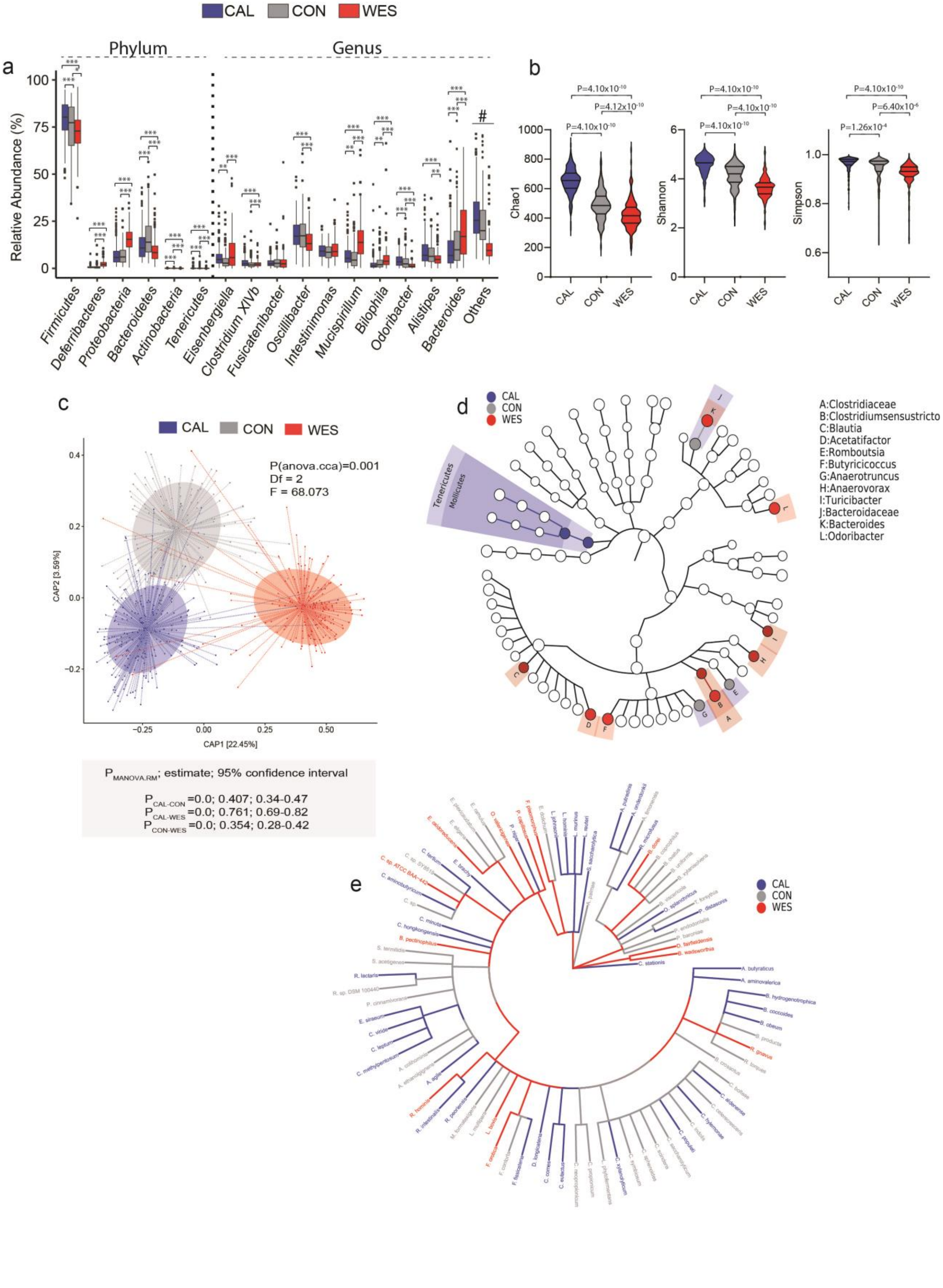
CAL
CON
WES
Phylum
Genus
a
Relative Abundance (%)
Firmicutes
Deferribacteres
Proteobacteria
Bacteroidetes
Actinobacteria
Tenericutes
Eisenbergiella
Clostridium XIVb
Fusicatenibacter
Oscillibacter
Intestinimonas
Mucispirillum
Bilophila
Odoribacter
Alistipes
Bacteroides
Others
b
Chao1
Shannon
Simpson
P=4.10x10-10
P=4.12x10-10
P=1.26x10-4
P=6.40x10-6
c
P(anova.cca)=0.001
Df = 2
F = 68.073
CAP1 [22.45%]
CAP2 [3.59%]
P MANOVA.RM; estimate; 95% confidence interval
P CAL-CON =0.0; 0.407; 0.34-0.47
P CAL-WES =0.0; 0.761; 0.69-0.82
P CON-WES =0.0; 0.354; 0.28-0.42
d
Tenericutes
Mollicutes
A:Clostridiaceae
B:Clostridiumsensustricto
C:Blautia
D:Acetatifactor
E:Romboutsia
F:Butyricicoccus
G:Anaerotruncus
H:Anaerovorax
I:Turicibacter
J:Bacteroidaceae
K:Bacteroides
L:Odoribacter
e

Supplementary Fig. 1. Diet modulates composition of bacterial active communities in the host. a. Relative abundance of bacterial phyla and genera in mice fed CAL (blue; n_{CAL}=240; n_{males}=109; $n_{females}$=131), CON (grey; n_{CON}=137; n_{males}=51; $n_{females}$=86), or WES diets (red; n_{CAL}=175; n_{males}=55; $n_{females}$=120). The band in the box plot indicates the median, the box indicates the first and third QRs, and the whiskers indicate 1.5*IQR. (*Firmicutes*: $P_{CAL-CON}$=0.0004, $P_{CAL-WES}$=$1.06x10^{-10}$, $P_{CON-WES}$=0.02; *Deferribacteres:* $P_{CAL-WES}$=$1.91x10^{-38}$, $P_{CON-WES}$=$1.08x10^{-36}$; *Proteobacteria:* $P_{CAL-WES}$=$2.14x10^{-37}$, $P_{CON-WES}$= $9.26x10^{-29}$;*Bacteroidetes:* $P_{CAL-CON}$=0.0004, $P_{CAL-WES}$=$1.63x10^{-5}$, $P_{CON-WES}$=$3.13x10^{-13}$; *Actinobacteria:* $P_{CAL-CON}$=0.002; $P_{CAL-WES}$=$7.96x10^{-33}$, $P_{CON-WES}$=$4.52x10^{-15}$; *Tenericutes:* $P_{CAL-WES}$=$1.63x10^{-5}$, $P_{CON-WES}$=0.0001; *Eisenbergiella*: $P_{CAL-CON}$=0.001, $P_{CON-WES}$= 0.0001; *Clostridium XlVb*: $P_{CAL-CON}$=0.0006, $P_{CAL-WES}$=0.02; *Oscillibacter:* $P_{CAL-WES}$=$4.51x10^{-5}$, $P_{CON-WES}$=0.0001; *Mucispirillum:* $P_{CAL-CON}$=0.005, $P_{CAL-WES}$=$2.67x10^{-23}$, $P_{CON-WES}$= $1.23x10^{-28}$; *Bilophila*: $P_{CAL-CON}$=0.005, $P_{CAL-WES}$=$1.42x10^{-18}$, $P_{CON-WES}$=$1.43x10^{-6}$; *Odoribacter:* $P_{CAL-CON}$=0.0005, $P_{CAL-WES}$=$7.53x10^{-17}$, $P_{CON-WES}$=0.0001; *Alistipes:* $P_{CAL-WES}$=$4.7x10^{-8}$; $P_{CON-WES}$=0.002; *Bacteroides:* $P_{CAL-CON}$=0.002, $P_{CAL-WES}$=$1.7x10^{-14}$, $P_{CON-WES}$= 0.0003). [#]Supplementary Data 6 shows statistical analysis of all taxa including low abundant taxa (“others”) across different diets. b. Violin plots showing alpha diversity indices of bacterial active communities in mice across different diets. The lines in the violin plot from bottom to top indicate 1st QR, median, and 3rd QR. The tips of the violin plot represent minima and maxima, and the width of the violin plot shows the frequency distribution of the data. c. Capscale plot of the BrayCurtis distance depicting beta diversity of active bacterial communities in mice across different diets. d. Differentially abundant microbial taxa (active communities) identified by the LEfSe algorithm in CAL (blue), CON (grey), and WES (red) mice. The root represents the fungal domain and the size of each node corresponds to the relative abundance of the taxon. e. Cladogram depicting bacterial indicator species among active communities for each diet. Statistical significance in panel a was determined using Kruskal–Wallis test followed by two-sided Mann–Whitney U test adjusted by FDR correction. In panel b statistical significance was determined using one-way ANOVA on residuals after sex and generation adjustment followed by Tukey’s multiple comparisons test. *Padj<0.05, **Padj<0.01, ***Padj<0.001. Data in panel d was analyzed using “anova.cca” function (999 permutations) followed by “MANOVA.RM” for post hoc analysis. Source data for a-d are provided as a Source Data file.

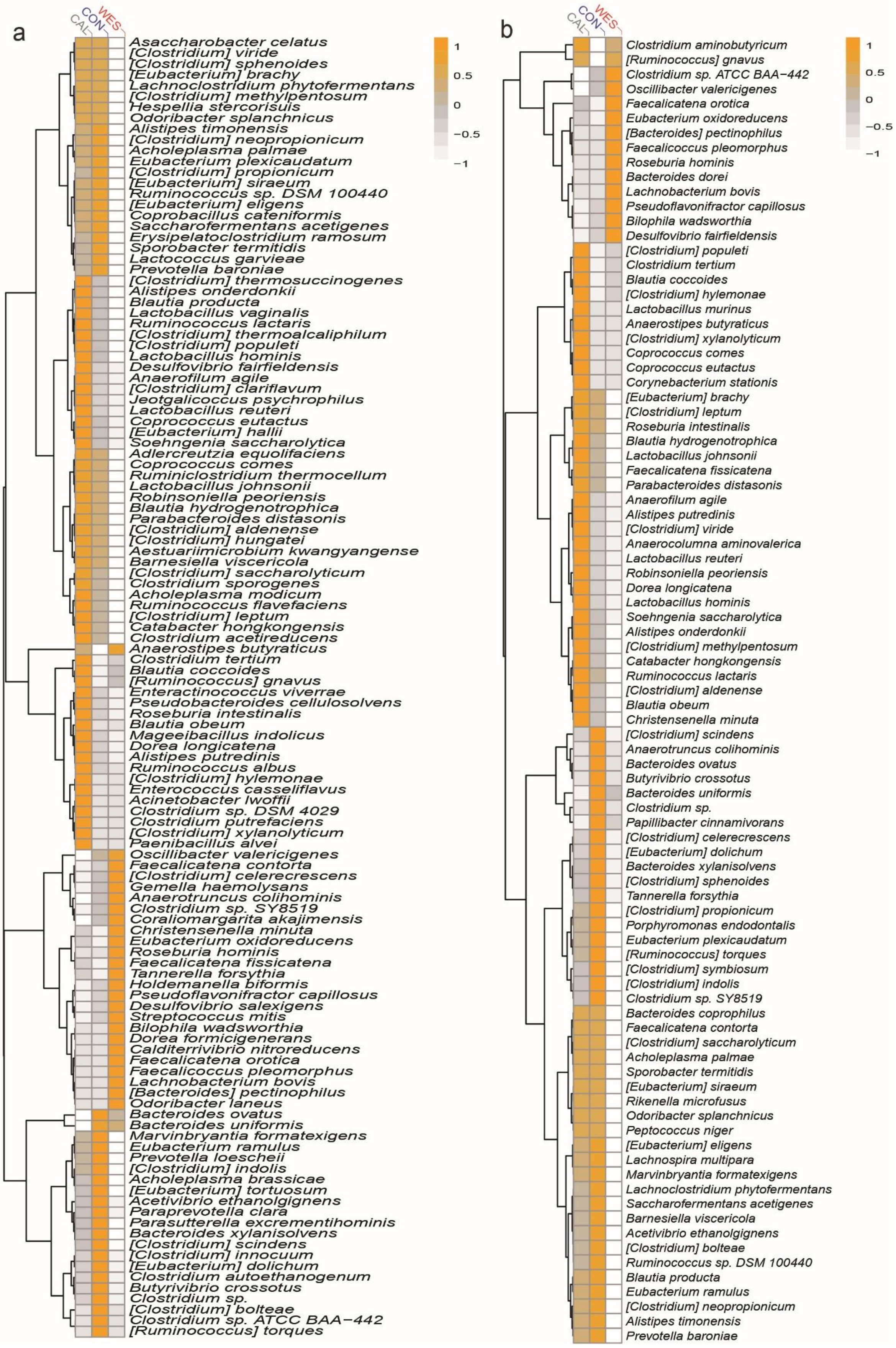
a
CAL
CON
WES
1
0.5
0
−0.5
−1
Asaccharobacter celatus
[Clostridium] viride
[Clostridium] sphenoides
[Eubacterium] brachy
Lachnoclostridium phytofermentans
[Clostridium] methylpentosum
Hespellia stercorisuis
Odoribacter splanchnicus
Alistipes timonensis
[Clostridium] neopropionicum
Acholeplasma palmae
Eubacterium plexicaudatum
[Clostridium] propionicum
[Eubacterium] siraeum
Ruminococcus sp. DSM 100440
[Eubacterium] eligens
Coprobacillus cateniformis
Saccharofermentans acetigenes
Erysipelatoclostridium ramosum
Sporobacter termitidis
Lactococcus garvieae
Prevotella baroniae
[Clostridium] thermosuccinogenes
Alistipes onderdonkii
Blautia producta
Lactobacillus vaginalis
Ruminococcus lactaris
[Clostridium] thermoalcaliphilum
[Clostridium] populeti
Lactobacillus hominis
Desulfovibrio fairfieldensis
Anaerofilum agile
[Clostridium] clariflavum
Jeotgalicoccus psychrophilus
Lactobacillus reuteri
Coprococcus eutactus
[Eubacterium] hallii
Soehngenia saccharolytica
Adlercreutzia equolifaciens
Coprococcus comes
Ruminiclostridium thermocellum
Lactobacillus johnsonii
Robinsoniella peoriensis
Blautia hydrogenotrophica
Parabacteroides distasonis
[Clostridium] aldenense
[Clostridium] hungatei
Aestuariimicrobium kwangyangense
Barnesiella viscericola
[Clostridium] saccharolyticum
Clostridium sporogenes
Acholeplasma modicum
Ruminococcus flavefaciens
[Clostridium] leptum
Catabacter hongkongensis
Clostridium acetireducens
Anaerostipes butyraticus
Clostridium tertium
Blautia coccoides
[Ruminococcus] gnavus
Enteractinococcus viverrae
Pseudobacteroides cellulosolvens
Roseburia intestinalis
Blautia obeum
Mageeibacillus indolicus
Dorea longicatena
Alistipes putredinis
Ruminococcus albus
[Clostridium] hylemonae
Enterococcus casseliflavus
Acinetobacter lwoffii
Clostridium sp. DSM 4029
Clostridium putrefaciens
[Clostridium] xylanolyticum
Paenibacillus alvei
Oscillibacter valericigenes
Faecalicatena contorta
[Clostridium] celerecrescens
Gemella haemolysans
Anaerotruncus colihominis
Clostridium sp. SY8519
Coraliomargarita akajimensis
Christensenella minuta
Eubacterium oxidoreducens
Roseburia hominis
Faecalicatena fissicatena
Tannerella forsythia
Holdemanella biformis
Pseudoflavonifractor capillosus
Desulfovibrio salexigens
Streptococcus mitis
Bilophila wadsworthia
Dorea formicigenerans
Calditerrivibrio nitroreducens
Faecalicatena orotica
Faecalicoccus pleomorphus
Lachnobacterium bovis
[Bacteroides] pectinophilus
Odoribacter laneus
Bacteroides ovatus
Bacteroides uniformis
Marvinbryantia formatexigens
Eubacterium ramulus
Prevotella loescheii
[Clostridium] indolis
Acholeplasma brassicae
[Eubacterium] tortuosum
Acetivibrio ethanolgignens
Paraprevotella clara
Parasutterella excrementihominis
Bacteroides xylanisolvens
[Clostridium] scindens
[Clostridium] innocuum
[Eubacterium] dolichum
Clostridium autoethanogenum
Butyrivibrio crossotus
Clostridium sp.
[Clostridium] bolteae
Clostridium sp. ATCC BAA−442
[Ruminococcus] torques
b
CAL
CON
WES
1
0.5
0
−0.5
−1
Clostridium aminobutyricum
[Ruminococcus] gnavus
Clostridium sp. ATCC BAA−442
Oscillibacter valericigenes
Faecalicatena orotica
Eubacterium oxidoreducens
[Bacteroides] pectinophilus
Faecalicoccus pleomorphus
Roseburia hominis
Bacteroides dorei
Lachnobacterium bovis
Pseudoflavonifractor capillosus
Bilophila wadsworthia
Desulfovibrio fairfieldensis
[Clostridium] populeti
Clostridium tertium
Blautia coccoides
[Clostridium] hylemonae
Lactobacillus murinus
Anaerostipes butyraticus
[Clostridium] xylanolyticum
Coprococcus comes
Coprococcus eutactus
Corynebacterium stationis
[Eubacterium] brachy
[Clostridium] leptum
Roseburia intestinalis
Blautia hydrogenotrophica
Lactobacillus johnsonii
Faecalicatena fissicatena
Parabacteroides distasonis
Anaerofilum agile
Alistipes putredinis
[Clostridium] viride
Anaerocolumna aminovalerica
Lactobacillus reuteri
Robinsoniella peoriensis
Dorea longicatena
Lactobacillus hominis
Soehngenia saccharolytica
Alistipes onderdonkii
[Clostridium] methylpentosum
Catabacter hongkongensis
Ruminococcus lactaris
[Clostridium] aldenense
Blautia obeum
Christensenella minuta
[Clostridium] scindens
Anaerotruncus colihominis
Bacteroides ovatus
Butyrivibrio crossotus
Bacteroides uniformis
Clostridium sp.
Papillibacter cinnamivorans
[Clostridium] celerecrescens
[Eubacterium] dolichum
Bacteroides xylanisolvens
[Clostridium] sphenoides
Tannerella forsythia
[Clostridium] propionicum
Porphyromonas endodontalis
Eubacterium plexicaudatum
[Ruminococcus] torques
[Clostridium] symbiosum
[Clostridium] indolis
Clostridium sp. SY8519
Bacteroides coprophilus
Faecalicatena contorta
[Clostridium] saccharolyticum
Acholeplasma palmae
Sporobacter termitidis
[Eubacterium] siraeum
Rikenella microfusus
Odoribacter splanchnicus
Peptococcus niger
[Eubacterium] eligens
Lachnospira multipara
Marvinbryantia formatexigens
Lachnoclostridium phytofermentans
Saccharofermentans acetigenes
Barnesiella viscericola
Acetivibrio ethanolgignens
[Clostridium] bolteae
Ruminococcus sp. DSM 100440
Blautia producta
Eubacterium ramulus
[Clostridium] neopropionicum
Alistipes timonensis
Prevotella baroniae

Supplementary Fig. 2. Indicator species analysis of bacterial communities in the gut of AIL mice across different dietary groups. a. Indicator species of bacterial standing communities (DNA). b. Indicator species analysis of bacterial active communities (RNA). Indicator species analysis in a and b was performed using ‘multipatt’ function of indicspecies R package. The ‘multipatt’ function uses the IndVal index (func = "IndVal.g") as test statistic with n=999 permutations. Source data for a-b are provided as a Source Data file. CAL, calorie-restricted diet; CON, control diet; WES, western diet.

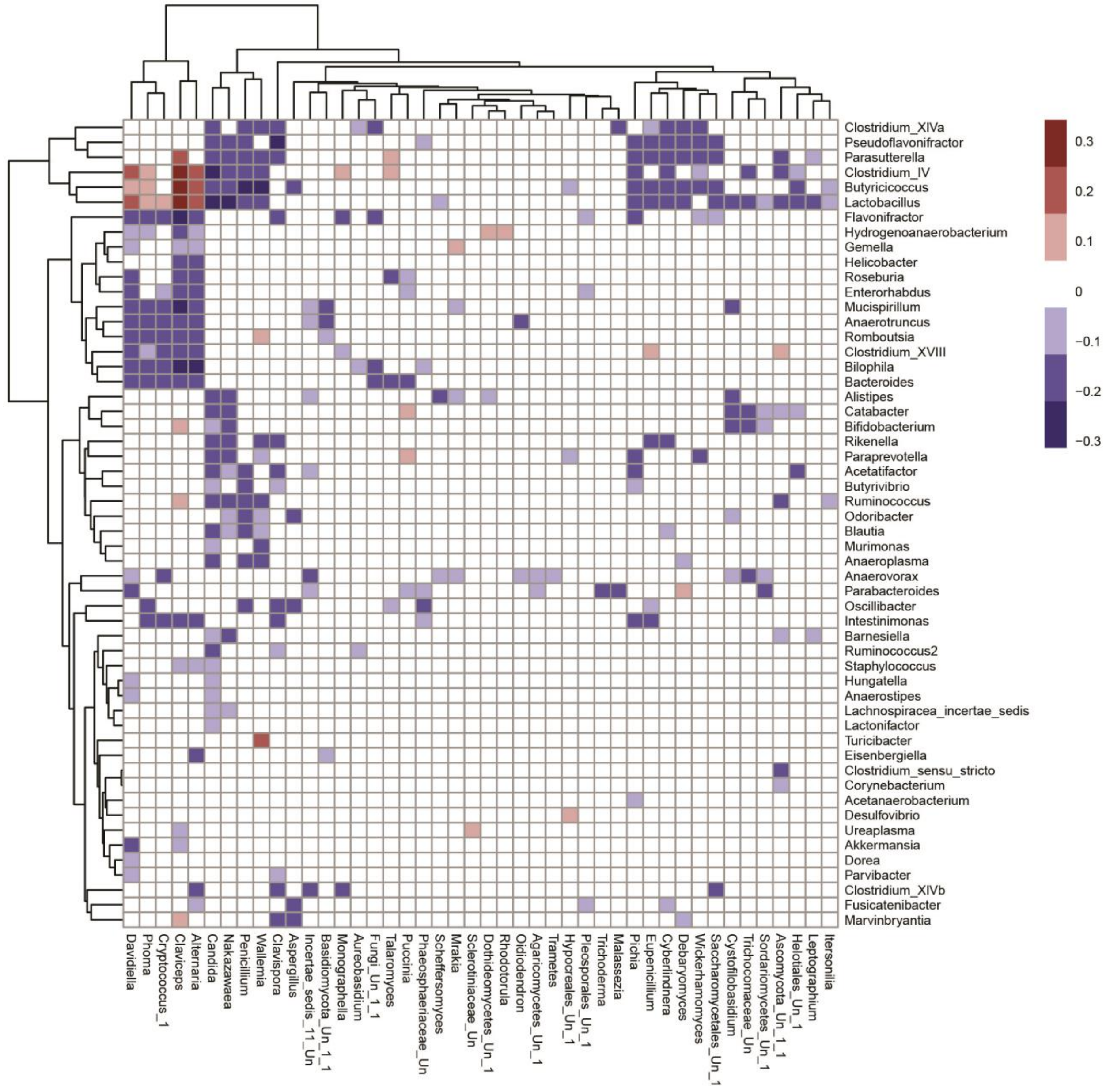

Supplementary Fig. 3. Interaction network between microbial active communities and fungi in the gut of AIL mice. Heatmap demonstrating significant (Padj<0.05) correlations between fungal genera (columns) abundances and bacterial genera (rows) abundances (active communities). The color codes of the cells indicate either positive (purple) or negative (orange) correlations among the species (Padj< 0.05); n=420 samples were used for the correlation analysis. Data were calculated using FastSpar implementation of the FastCC algorithm (999 permutations). P-value were adjusted using Benjamini-Hochberg correction. Source data are provided as a Source Data file.

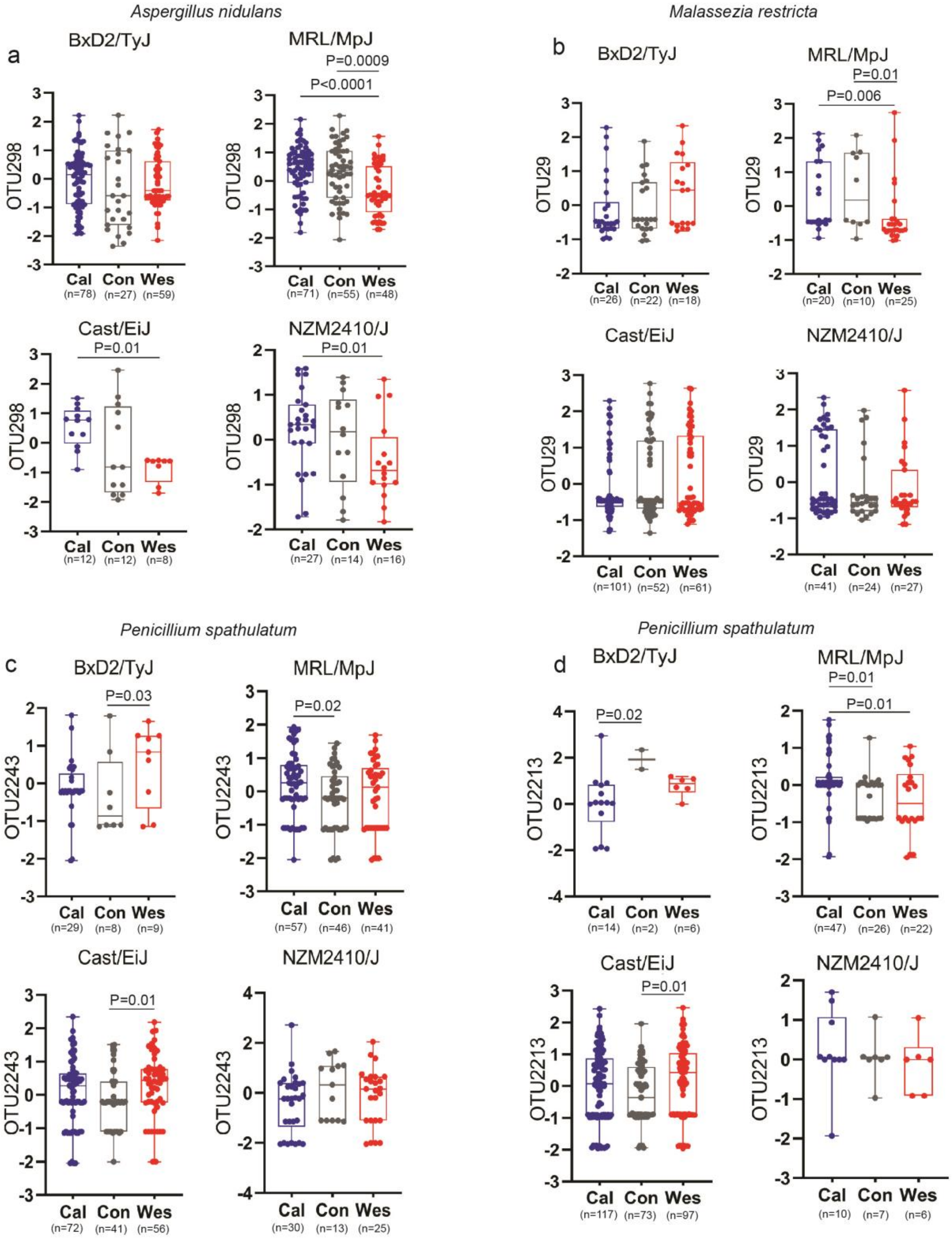

Aspergillus nidulans
a
BxD2/TyJ
MRL/MpJ
P=0.0009
P<0.0001
OTU298
Cal Con Wes
(n=78) (n=27) (n=59)
(n=71) (n=55) (n=48)
Cast/EiJ
P=0.01
NZM2410/J
P=0.01
(n=12) (n=12) (n=8)
(n=27) (n=14) (n=16)
Malassezia restricta
b
BxD2/TyJ
MRL/MpJ
P=0.01
P=0.006
OTU29
(n=26) (n=22) (n=18)
(n=20) (n=10) (n=25)
Cast/EiJ
NZM2410/J
(n=101) (n=52) (n=61)
(n=41) (n=24) (n=27)
Penicillium spathulatum
c
BxD2/TyJ
P=0.03
MRL/MpJ
P=0.02
OTU2243
(n=29) (n=8) (n=9)
(n=57) (n=46) (n=41)
Cast/EiJ
P=0.01
NZM2410/J
(n=72) (n=41) (n=56)
(n=30) (n=13) (n=25)
Penicillium spathulatum
d
BxD2/TyJ
P=0.02
MRL/MpJ
P=0.01
P=0.01
OTU2213
(n=14) (n=2) (n=6)
(n=47) (n=26) (n=22)
Cast/EiJ
P=0.01
NZM2410/J
(n=117) (n=73) (n=97)
(n=10) (n=7) (n=6)

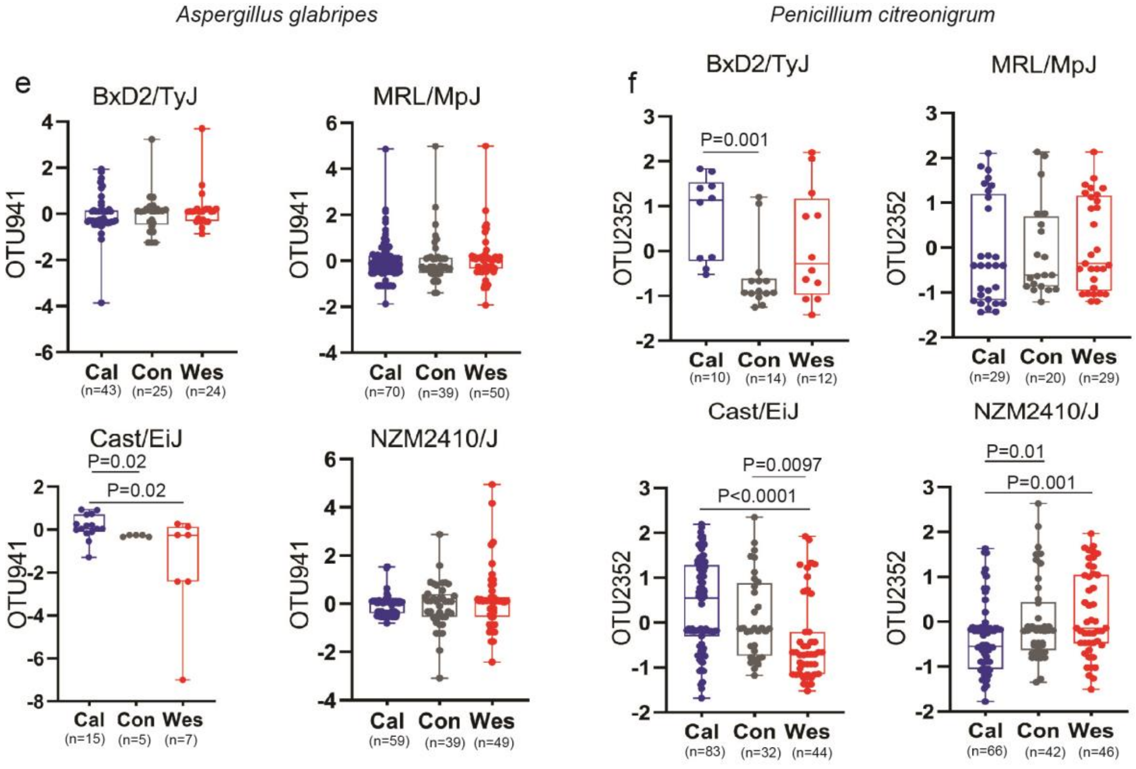

Supplementary Fig. 4. Effect of gene diet interaction on the composition of fungi species in the gut in AIL mice. Association of alleles within the peak SNP of the identified IntDiet QTL with the standardized residuals of fungi species across individual diets. The x-axis describes the individual diets, y-axis shows residuals (as phenotype) after regressing taxa (VST transformed) against confounding factors such as cage, generation, and sex. Each figure consists of four plots, which depict the information for the individual founder strain. The individual mice were assigned to founder strain, based on maximum posterior probability calculation at the peak SNP. Statistical significance was assessed using Kruskal-Wallis test followed by FDR correction for multiple comparisons. Individual P values for significant comparisons are indicated in the figure and adjusted q value are provided in figure legend as follows. a. Association of alleles at the marker UNC30601828 on Chr 19 (mm10) with the distribution of *Aspergillus nidulans.* (MRL/MpJ: $q_{Cal-Wes}$=0.0001, $q_{Con-Wes}$=0.0013; CAST/EiJ: $q_{Cal-Wes}$=0.04; NZM2410/J: $q_{Cal-Wes}$=0.05) b. Association of alleles at the UNC080619407 marker on Chr 8 with the distribution of *Malassezia restricta* (MRL/MpJ: $q_{Cal-Wes}$=0.01, $q_{Wes-Con}$=0.02). Association of alleles at the marker UNC29116030 (MRL/MpJ: $q_{Cal-Con}$=0.07; BXD2: $q_{Wes-Con}$=0.1; CAST/EiJ: $q_{Con-Wes}$=0.03) (c) and its nearby marker UNC29082687 (MRL/MpJ: $q_{Cal-Con}$=0.02, $q_{Cal-Wes}$=0.02; BXD2: $q_{Cal-Con}$=0.06; CAST/EiJ: $q_{Con-Wes}$=0.05) (d) on Chr 18 with the distribution of *Penicillium spathulatum.* Association of alleles at the marker UNC200105170 (Chr X) and marker CEAJAX00009745 (Chr 1) on the distribution of *Aspergillus glabripes* (CAST/EiJ: $q_{Con-Cal}$=0.03, $q_{Wes-Cal}$=0.03) (e) and *Penicillium citreonigrum* (BXD2: $q_{Cal-Con}$=0.003; CAST/EiJ: $q_{Con-Wes}$=0.01, $q_{Wes-Cal}$<0.0001; NZM2410/J: $q_{Cal-Wes}$=0.0042, $q_{Cal-Con}$=0.01) (f), respectively. The band in the box plot indicates the median, the box indicates the first and third QRs, and the whiskers indicate 1.5*IQR. Number of mice/group used to generate the plot is indicated in the brackets

below the x-axis of each individual plot. The individual values are indicated as dots in the plot. Source data for a-f are provided as a Source Data file. Cal, calorie-restricted diet; Con, control diet; Wes, western diet.
