# Supplementary material for: Impact of diet and host genetics on the murine intestinal mycobiome
Source: Nat Commun. 2023 Feb 14;14:834. doi: 10.1038/s41467-023-36479-z (PMC9929102; doi:10.1038/s41467-023-36479-z)
Supplement: Supplementary file 3 — Description of Additional Supplementary Files [file 41467_2023_36479_MOESM3_ESM.pdf]

Description of Additional Supplementary Files

File Name: Supplementary Data 1

Description: Mouse cohort and samples used for downstream analyses in this study.

File Name: Supplementary Data 2

Description: Nominal and adjusted P-values across different covariates for fungal taxa. Data was statistically assessed using Kruskal–Wallis test followed by two-sided Mann–Whitney U test adjusted by FDR correction. Related to Figure 1.

File Name: Supplementary Data 3

Description: OTUs table of identified fungi across all sequenced samples. Related to Figure 1.

File Name: Supplementary Data 4

Description: Indicator species for fungal OTUs across different dietary regimens. Indicator species analysis was performed using 'multipatt' function of indicspecies R package. The 'multipatt' function uses the IndVal index (func = "IndVal.g") as test statistic with n=999 permutations. Related to Figure 1.

File Name: Supplementary Data 5

Description: Nominal and adjusted P-values across different covariates for bacterial taxa (standing communities). Data was statistically assessed using Kruskal–Wallis test followed by two-sided Mann–Whitney U test adjusted by FDR correction. Related to Figure 2.

File Name: Supplementary Data 6

Description: Nominal and adjusted P-values across different covariates for bacterial taxa (active communities). Data was statistically assessed using Kruskal–Wallis test followed by two-sided Mann–Whitney U test adjusted by FDR correction. Related to Supplementary Figure 1.

File Name: Supplementary Data 7

Description: OTUs table of identified bacterial standing communities across all sequenced samples. Related to Figure 2.

File Name: Supplementary Data 8

Description: OTUs table of identified bacterial active communities across all sequenced samples. Related to Supplementary Figure 1.

File Name: Supplementary Data 9

Description: Indicator species for bacterial standing communities across different dietary regimens. Indicator species analysis was performed using 'multipatt' function of indicspecies R package. The 'multipatt' function uses the IndVal index (func = "IndVal.g") as test statistic with n=999 permutations. Related to Figure 2.

File Name: Supplementary Data 10

Description: Indicator species for bacterial active communities across different dietary regimens. Indicator species analysis was performed using 'multipatt' function of indicspecies R package. The 'multipatt' function uses the IndVal index (func = "IndVal.g") as test statistic with n=999 permutations. Related to Supplementary Figure 2.

File Name: Supplementary Data 11

Description: Correlations between fungi and standing bacterial communities. Correlation coefficient was calculated using FastSpar that uses the SparCC algorithm with n=999 permutations. P-value were adjusted using Benjamini-Hochberg correction. Related to Figure 3.

File Name: Supplementary Data 12

Description: Correlations between fungi and active bacterial communities. Correlation coefficient was calculated using FastSpar that uses the SparCC algorithm with n=999 permutations. P-value were adjusted using Benjamini-Hochberg correction. Related to Supplementary Figure 3.

File Name: Supplementary Data 13

Description: Bacterial QTL and the identified candidate genes. The table shows all mapped QTL derived from bacterial DNA and RNA sequences across different models Add (host genetics-mycobiome interactions only), IntDiet (host genetics-diet interactions only), and IntSex (host genetics-sex interactions only) with taxonomical assignment of bacteria from phylum to species level on chromosomal loci. Percentages of phenotypic variation (h2) for each QTL explained by sex, generation (Gen), diet, and cage are indicated as %h2 LOD, %h2 Sex, %h2 Gen, %h2 Diet, and % h2 Cage, respectively. Also, spearman's correlation coefficients are displayed as ρ (sex), ρ (Diet) and ρ (Gen). Related to Figure 4.

File Name: Supplementary Data 14

Description: List of previously published bacterial QTL and overlap with QTL identified in our study.

File Name: Supplementary Data 15

Description: List of all primer sequences that were used for 16S rRNA and ITS2 sequencing in our study.
