# Supplementary material for: Impact of diet and host genetics on the murine intestinal mycobiome
Source: Nat Commun. 2023 Feb 14;14:834. doi: 10.1038/s41467-023-36479-z (PMC9929102; doi:10.1038/s41467-023-36479-z)
Supplement: Supplementary file 18 — Supplementary Dataset 15 [file 41467_2023_36479_MOESM18_ESM.docx]

Supplementary Data 15. List of all primer sequences that were used for 16S rRNA and ITS2 sequencing in our study.

**Name of the primer Sequence (5'-3')**

**16S rRNA forward primers**

27F-MID-1 AATGATACGGCGACCACCGAGATCTACACAACCGCATTATGGTAATTGTAGAGTTTGATCCTGGCTCAG

27F-MID-2 AATGATACGGCGACCACCGAGATCTACACAAGGCCTTTATGGTAATTGTAGAGTTTGATCCTGGCTCAG

27F-MID-3 AATGATACGGCGACCACCGAGATCTACACAGAGTGTGTATGGTAATTGTAGAGTTTGATCCTGGCTCAG

27F-MID-4 AATGATACGGCGACCACCGAGATCTACACCACAAGTCTATGGTAATTGTAGAGTTTGATCCTGGCTCAG

27F-MID-5 AATGATACGGCGACCACCGAGATCTACACCGTTCCTATATGGTAATTGTAGAGTTTGATCCTGGCTCAG

27F-MID-6 AATGATACGGCGACCACCGAGATCTACACGCTTGGATTATGGTAATTGTAGAGTTTGATCCTGGCTCAG

27F-MID-7 AATGATACGGCGACCACCGAGATCTACACGTCAACACTATGGTAATTGTAGAGTTTGATCCTGGCTCAG

27F-MID-8 AATGATACGGCGACCACCGAGATCTACACGTCACTGATATGGTAATTGTAGAGTTTGATCCTGGCTCAG

27F-MID-9 AATGATACGGCGACCACCGAGATCTACACTCTCGTCATATGGTAATTGTAGAGTTTGATCCTGGCTCAG

27F-MID-10 AATGATACGGCGACCACCGAGATCTACACTTGGTACGTATGGTAATTGTAGAGTTTGATCCTGGCTCAG

27F-MID-11 AATGATACGGCGACCACCGAGATCTACACCGTTGGATTATGGTAATTGTAGAGTTTGATCCTGGCTCAG

27F-MID-12 AATGATACGGCGACCACCGAGATCTACACCGTTAAGCTATGGTAATTGTAGAGTTTGATCCTGGCTCAG

27F-MID-13 AATGATACGGCGACCACCGAGATCTACACACAGCTCATATGGTAATTGTAGAGTTTGATCCTGGCTCAG

27F-MID-14 AATGATACGGCGACCACCGAGATCTACACGACAAGTGTATGGTAATTGTAGAGTTTGATCCTGGCTCAG

27F-MID-15 AATGATACGGCGACCACCGAGATCTACACGCATTAGCTATGGTAATTGTAGAGTTTGATCCTGGCTCAG

27F-MID-16 AATGATACGGCGACCACCGAGATCTACACTGTGGACTTATGGTAATTGTAGAGTTTGATCCTGGCTCAG

**16S rRNA reverse primers**

338R-MID-A CAAGCAGAAGACGGCATACGAGATAACCGGAAAGTCAGTCAGCCTGCTGCCTCCCGTAGGAGT

338R-MID-B CAAGCAGAAGACGGCATACGAGATAGAGTGACAGTCAGTCAGCCTGCTGCCTCCCGTAGGAGT

338R-MID-C CAAGCAGAAGACGGCATACGAGATCAACTGGTAGTCAGTCAGCCTGCTGCCTCCCGTAGGAGT

338R-MID-D CAAGCAGAAGACGGCATACGAGATCGTTCGTTAGTCAGTCAGCCTGCTGCCTCCCGTAGGAGT

338R-MID-E CAAGCAGAAGACGGCATACGAGATCTGTTCACAGTCAGTCAGCCTGCTGCCTCCCGTAGGAGT

338R-MID-F CAAGCAGAAGACGGCATACGAGATGCTTGCAAAGTCAGTCAGCCTGCTGCCTCCCGTAGGAGT

338R-MID-G CAAGCAGAAGACGGCATACGAGATGTCAACTGAGTCAGTCAGCCTGCTGCCTCCCGTAGGAGT

338R-MID-H CAAGCAGAAGACGGCATACGAGATTCCTCATGAGTCAGTCAGCCTGCTGCCTCCCGTAGGAGT

338R-MID-I CAAGCAGAAGACGGCATACGAGATTCGACTAGAGTCAGTCAGCCTGCTGCCTCCCGTAGGAGT

338R-MID-J CAAGCAGAAGACGGCATACGAGATTTGCAAGCAGTCAGTCAGCCTGCTGCCTCCCGTAGGAGT

338R-MID-K CAAGCAGAAGACGGCATACGAGATACACCTCTAGTCAGTCAGCCTGCTGCCTCCCGTAGGAGT

338R-MID-L CAAGCAGAAGACGGCATACGAGATATCGTAGCAGTCAGTCAGCCTGCTGCCTCCCGTAGGAGT

338R-MID-M CAAGCAGAAGACGGCATACGAGATCTCTTGACAGTCAGTCAGCCTGCTGCCTCCCGTAGGAGT

338R-MID-N CAAGCAGAAGACGGCATACGAGATCCTACCATAGTCAGTCAGCCTGCTGCCTCCCGTAGGAGT

338R-MID-O CAAGCAGAAGACGGCATACGAGATCTGAAGTCAGTCAGTCAGCCTGCTGCCTCCCGTAGGAGT

338R-MID-P CAAGCAGAAGACGGCATACGAGATACGATCGTAGTCAGTCAGCCTGCTGCCTCCCGTAGGAGT

338R-MID-Q CAAGCAGAAGACGGCATACGAGATATATGGCCAGTCAGTCAGCCTGCTGCCTCCCGTAGGAGT

338R-MID-R CAAGCAGAAGACGGCATACGAGATTTCGATGGAGTCAGTCAGCCTGCTGCCTCCCGTAGGAGT

338R-MID-S CAAGCAGAAGACGGCATACGAGATTACGTACGAGTCAGTCAGCCTGCTGCCTCCCGTAGGAGT

338R-MID-T CAAGCAGAAGACGGCATACGAGATGATCACGTAGTCAGTCAGCCTGCTGCCTCCCGTAGGAGT

338R-MID-U CAAGCAGAAGACGGCATACGAGATGTGACAGAAGTCAGTCAGCCTGCTGCCTCCCGTAGGAGT

338R-MID-V CAAGCAGAAGACGGCATACGAGATTGAGTGTCAGTCAGTCAGCCTGCTGCCTCCCGTAGGAGT

338R-MID-W CAAGCAGAAGACGGCATACGAGATGAGAAGAGAGTCAGTCAGCCTGCTGCCTCCCGTAGGAGT

338R-MID-X CAAGCAGAAGACGGCATACGAGATTCTGGACAAGTCAGTCAGCCTGCTGCCTCCCGTAGGAGT

**16S rRNA sequencing primers**

16SRead_1 TATGGTAATTGTAGAGTTTGATCCTGGCTCAG

16SRead_2 AGTCAGTCAGCCTGCTGCCTCCCGTAGGAGT

16SIndex ACTCCTACGGGAGGCAGCAGGCTGACTGACT

**ITS2 forward primers**

ITSF.SB501 AATGATACGGCGACCACCGAGATCTACACCTACTATATATGGTAATTGGTCCTCCGCTTATTGATATGC

ITSF.SB502 AATGATACGGCGACCACCGAGATCTACACCGTTACTATATGGTAATTGGTCCTCCGCTTATTGATATGC

ITSF.SB503 AATGATACGGCGACCACCGAGATCTACACAGAGTCACTATGGTAATTGGTCCTCCGCTTATTGATATGC

ITSF.SB504 AATGATACGGCGACCACCGAGATCTACACTACGAGACTATGGTAATTGGTCCTCCGCTTATTGATATGC

ITSF.SB505 AATGATACGGCGACCACCGAGATCTACACACGTCTCGTATGGTAATTGGTCCTCCGCTTATTGATATGC

ITSF.SB506 AATGATACGGCGACCACCGAGATCTACACTCGACGAGTATGGTAATTGGTCCTCCGCTTATTGATATGC

ITSF.SB507 AATGATACGGCGACCACCGAGATCTACACGATCGTGTTATGGTAATTGGTCCTCCGCTTATTGATATGC

ITSF.SB508 AATGATACGGCGACCACCGAGATCTACACGTCAGATATATGGTAATTGGTCCTCCGCTTATTGATATGC

ITSF.SB509 AATGATACGGCGACCACCGAGATCTACACCTGAAGTCTATGGTAATTGGTCCTCCGCTTATTGATATGC

ITSF.SB510 AATGATACGGCGACCACCGAGATCTACACACGATCGTTATGGTAATTGGTCCTCCGCTTATTGATATGC

ITSF.SB511 AATGATACGGCGACCACCGAGATCTACACATATGGCCTATGGTAATTGGTCCTCCGCTTATTGATATGC

ITSF.SB512 AATGATACGGCGACCACCGAGATCTACACTTCGATGGTATGGTAATTGGTCCTCCGCTTATTGATATGC

ITSF.SB513 AATGATACGGCGACCACCGAGATCTACACTTGGTACGTATGGTAATTGGTCCTCCGCTTATTGATATGC

ITSF.SB514 AATGATACGGCGACCACCGAGATCTACACCGTTGGATTATGGTAATTGGTCCTCCGCTTATTGATATGC

ITSF.SB515 AATGATACGGCGACCACCGAGATCTACACCGTTAAGCTATGGTAATTGGTCCTCCGCTTATTGATATGC

ITSF.SB516 AATGATACGGCGACCACCGAGATCTACACACAGCTCATATGGTAATTGGTCCTCCGCTTATTGATATGC

ITSF.SB517 AATGATACGGCGACCACCGAGATCTACACGACAAGTGTATGGTAATTGGTCCTCCGCTTATTGATATGC

ITSF.SB518 AATGATACGGCGACCACCGAGATCTACACGCATTAGCTATGGTAATTGGTCCTCCGCTTATTGATATGC

ITSF.SB519 AATGATACGGCGACCACCGAGATCTACACTGTGGACTTATGGTAATTGGTCCTCCGCTTATTGATATGC

**ITS2 reverse primers**

ITSR.SA701 CAAGCAGAAGACGGCATACGAGATAACTCTCGAGTCAGTCAGCCGTGARTCATCGAATCTTTG

ITSR.SA702 CAAGCAGAAGACGGCATACGAGATACTATGTCAGTCAGTCAGCCGTGARTCATCGAATCTTTG

ITSR.SA703 CAAGCAGAAGACGGCATACGAGATAGTAGCGTAGTCAGTCAGCCGTGARTCATCGAATCTTTG

ITSR.SA704 CAAGCAGAAGACGGCATACGAGATCAGTGAGTAGTCAGTCAGCCGTGARTCATCGAATCTTTG

ITSR.SA705 CAAGCAGAAGACGGCATACGAGATCGTACTCAAGTCAGTCAGCCGTGARTCATCGAATCTTTG

ITSR.SA706 CAAGCAGAAGACGGCATACGAGATCTACGCAGAGTCAGTCAGCCGTGARTCATCGAATCTTTG

ITSR.SA707 CAAGCAGAAGACGGCATACGAGATGGAGACTAAGTCAGTCAGCCGTGARTCATCGAATCTTTG

ITSR.SA708 CAAGCAGAAGACGGCATACGAGATGTCGCTCGAGTCAGTCAGCCGTGARTCATCGAATCTTTG

ITSR.SA709 CAAGCAGAAGACGGCATACGAGATGTCGTAGTAGTCAGTCAGCCGTGARTCATCGAATCTTTG

ITSR.SA710 CAAGCAGAAGACGGCATACGAGATTAGCAGACAGTCAGTCAGCCGTGARTCATCGAATCTTTG

ITSR.SA711 CAAGCAGAAGACGGCATACGAGATTCATAGACAGTCAGTCAGCCGTGARTCATCGAATCTTTG

ITSR.SA712 CAAGCAGAAGACGGCATACGAGATTCGCTATAAGTCAGTCAGCCGTGARTCATCGAATCTTTG

ITSR.SA713 CAAGCAGAAGACGGCATACGAGATTACGTACGAGTCAGTCAGCCGTGARTCATCGAATCTTTG

ITSR.SA714 CAAGCAGAAGACGGCATACGAGATGATCACGTAGTCAGTCAGCCGTGARTCATCGAATCTTTG

ITSR.SA715 CAAGCAGAAGACGGCATACGAGATGTGACAGAAGTCAGTCAGCCGTGARTCATCGAATCTTTG

ITSR.SA716 CAAGCAGAAGACGGCATACGAGATAACCGGAAAGTCAGTCAGCCGTGARTCATCGAATCTTTG

ITSR.SA717 CAAGCAGAAGACGGCATACGAGATCAACTGGTAGTCAGTCAGCCGTGARTCATCGAATCTTTG

ITSR.SA718 CAAGCAGAAGACGGCATACGAGATCGTTCGTTAGTCAGTCAGCCGTGARTCATCGAATCTTTG

ITSR.SA719 CAAGCAGAAGACGGCATACGAGATCTGTTCACAGTCAGTCAGCCGTGARTCATCGAATCTTTG

ITSR.SA720 CAAGCAGAAGACGGCATACGAGATGCTTGCAAAGTCAGTCAGCCGTGARTCATCGAATCTTTG

ITSR.SA721 CAAGCAGAAGACGGCATACGAGATGTCAACTGAGTCAGTCAGCCGTGARTCATCGAATCTTTG

ITSR.SA722 CAAGCAGAAGACGGCATACGAGATTCCTCATGAGTCAGTCAGCCGTGARTCATCGAATCTTTG

ITSR.SA723 CAAGCAGAAGACGGCATACGAGATTTGCAAGCAGTCAGTCAGCCGTGARTCATCGAATCTTTG

ITSR.SA724 CAAGCAGAAGACGGCATACGAGATACACCTCTAGTCAGTCAGCCGTGARTCATCGAATCTTTG

**ITS2 sequencing primers**

ITSRead_1 TATGGTAATTGGTCCTCCGCTTATTGATATGC

ITSRead_2 AGTCAGTCAGCCGTGARTCATCGAATCTTTG

ITSIndex CAAAGATTCGATGARTCACGGCTGACTGACT
